# Profiling of seminal plasma proteins to identify the fertility of Simmental bull with low semen quality

**DOI:** 10.5455/javar.2023.j689

**Published:** 2023-09-24

**Authors:** Abdullah Baharun, Annisa Rahmi, Dede Kardaya, Syahruddin Said, Mokhamad Fahrudin, Raden Iis Arifiantini, Ni Wayan Kurniani Karja

**Affiliations:** 1 Department of Animals Science, Faculty of Agriculture, Djuanda University, Bogor, Indonesia; 2 Research Center for Applied Zoology, National Research and Innovation Agency, Bogor, Indonesia; 3 Division of Anatomy, Histology and Embryology, School of Veterinary Medicine and BiomedicalSciences, IPB University, Bogor, Indonesia; 4 Division of Veterinary Reproduction and Obstetrics, School of Veterinary Medicine and Biomedical Sciences, IPB University, Bogor, Indonesia

**Keywords:** Fresh semen, proteome, Simmental bull proteomic, seminal plasma

## Abstract

**Objective:**

The present study analyzed the seminal plasma proteome and possible relationships between proteins and semen quality in azoospermic and normal Simmental bulls.

**Materials and Methods:**

Fresh semen plasma samples from the Lembang Artificial Insemination Center were used for this study, including one bull (76´ ejaculate) with very poor semen quality/azoospermia (poor fresh semen/infertile; PFS) and three bulls with normal semen quality (normal fresh semen; NFS) for proteomic analysis using a pooled system (NFS-Stud) (60´ ejaculate). The only males obtained with very low quality or azoospermia (PFS) had sperm motility of <10% (one head). Bulls with azoospermic conditions produce fresh semen without sperm or with very little sperm concentration. A total of 109 proteins were identified in the seminal plasma of Simmental bulls analyzed using liquid chromatography-mass spectrometry. Bioinformatics analysis was used to explore total protein, expression, function, and protein mechanism in the seminal plasma of Simmental bulls.

**Results:**

The results showed that the seminal plasma proteins expressed in NFS bulls include ELSPBP1, SIL1, HSPA13, angiotensin-1 covering enzyme, and CRISP1. On the other hand, B2M, C3, CFB, venin-2, and cathepsin S contribute significantly to PFS. The NFS bull proteins play important roles in sperm capacitation, protein transport, sperm motility, spermatogenesis, immune tolerance, and fertilization, while the PFS proteins perform apoptotic and antigen pathway functions.

**Conclusion:**

There is an interaction between proteins in the seminal plasma of males with poor semen quality (PFS) and cases of infertility (azoospermia) that cause a decrease in sperm quality in PFS bulls.

## Introduction

The success of reproductive efficiency in livestock is reflected in reproductive health, which may indicate a high fertilization rate. The determining factor for successful fertilization is the intrinsic and extrinsic factors of sperm, namely sperm plasma, which is associated with male fertility and plays an important role in determining the ability of sperm to fertilize oocytes [1]. Sperm fertility is the capability of sperm to fertilize and activate oocytes to support embryonic development. Both are essential factors that can affect male fertility [2]. The reproductive selection of bull fertility by the breeding soundness examination (BSE) method shows that the production and quality of sperm produced are not optimal, although bulls have passed BSE selection, and sperm concentration and motility are even very low [3]. Several studies have shown that age [3], individual differences [4], environmental factors [5], and seminal plasma and sperm protein expression may have an influence on sperm quality and fertility [1]. Protein, a secretory component of sperm, is one of the factors that can influence sperm physiology for normal fertilization [6].

Expression of specific sperm plasma proteins allows males with normal libido to have low sperm quality. Sperm seminal plasma proteins play critical roles in acrosome capacitation, response fertilization, and sperm-ovum interactions [1]. Diansyah et al. [7] reported that interactions between different sperm plasma proteins can have negative effects on sperm quality, such as low sperm motility and sperm concentration, which can even lead to ejaculation without sperm [8]. The decreased motility and sperm concentration in fresh semen may be related to the immunogenic or autoimmune system emanating from the animals, especially from sperm plasma and testis [9]. Sperm protection from the autoimmune system begins in the testes and depends on the presence of the blood-testicular barrier (BTB), which protects sperm during the stages of germ cell development and spermiogenesis [10], which is related to sperm quality. There is a correlation between sperm quality and BTB, including peptidyl-prolyl cis/trans isomerase, the Fas ligand system, toll-like receptors, and cytokines that can affect semen quality, regulation of the immune system in sperm [9], and male fertility [11].

There are no reports on the physiological aspects affecting the immune response and infertility and their relationship with sperm quality and fertility through the biological roles and functions of bovine sperm plasma proteins. Currently, there are new developments in the evaluation of male fertility based on proteomics analysis, a molecular analysis based on functions, biological processes, pathways, and the interaction of plasma protein expression in semen that determines fertility and semen quality. Thus, the objective of this study is to identify proteins in seminal plasma associated with infertility cases as part of Indonesian producers' efforts to increase the reproductive performance of Simmental bulls.

## Materials and Methods

### Ethical approval

The IPB University Animal Ethics Committee approved the animal models and experimental designs for this study with certificate number 158-2019 IPB.

### Seminal plasma collection and preparation

Fresh semen plasma samples from the Lembang Artificial Insemination Center (AIC) were used for this study, including one bull (76´ ejaculate) with very poor semen quality/azoospermia (poor fresh semen/infertile; PFS) and three bulls with normal semen quality (normal fresh semen; NFS) for proteomic analysis using a pooled system (NFS-Stud) (60´ ejaculate). The only males obtained with very low quality, or azoospermia (PFS), had sperm motility of <10% (one head). Bulls with azoospermic conditions produce fresh semen without sperm or with very little sperm concentration. Fresh sperm criteria based on secondary sperm motility data with values of ≥70% and <10% belong to Lembang AIC in 2018–2019.

Semen collection was performed from 7:00 a.m. to 9:00 a.m. using an artificial vagina. Seminal plasma was obtained by centrifuging 2 ml of fresh semen from NFS and PFS Simmental bulls for 30 min at 6,500 × rpm. The supernatant and pellet were stored at −20^°^C for further analysis.

### Protein quantification

The determination of seminal plasma protein concentration was performed according to the protocol of the Coomasie User Guide (Bradford) Protein Assay Kit. The data obtained were analyzed using the Thermo Skanlt RE software for Multiskan Go Software version 3.2.

A total of 25 gm of aliquoted seminal plasma protein in a microtube and dried in a vacuum. Samples were lysed with a buffer containing 8 M urea, 0.02 M triethylammonium bicarbonate (TEAB), and 0.5 M dithiothreitol (DTT), followed by incubation at 55^°^C and shaking at 400 rpm (Eppendorf^®^ Thermomixer^®^ R, Sigma-Aldrich, Darmstadt, Germany) for 25 min. The alkylation process was then performed by adding iodoacetamide to a final concentration of 0.014 M. The mixture was kept dark at 21^°^C and 400 rpm for 40 min. The digestion process was done by adding 0.005 M DTT, 0.001 M CaCl_2_, and 0.02 M TEAB until the final volume was 75 µl. Trypsin enzyme (Promega, Fitchburg, WI) with an enzyme/substrate ratio of 1:50 (w/w) was used as the enzyme for the digestion process and incubated at 37^°^C for 18 h. To stop trypsin activity, 1% of the final volume of trifluoroacetic acid solution was added to the reaction [6]. The purification was performed with C18 spin columns, each of which had a porous C18 reserved-phase resin that could bind peptides well.

### Peptide fractionation and liquid chromatography-mass spectrometry (LC-MS/MS) analysis

Peptides from each sample were analyzed using LC-MS/MS on the Nano LC Ultimate 3000 Series System Tandem Q Exactive Plus Orbitrap HRMS (Thermo Scientific) system with a PepMap RSLC C18 column (75 µm inner diameter, 15 cm, 3 µm, 100 pore size, part number ES 800 (Thermo Scientific) at a flow rate of 300 ml/min. Elution of peptides from the column gradient solvent B for 0–3 min (0.1% formic acid, 98% acetonitrile); 2%–35% solvent B for 3–30 min, 35%–90% solvent B for 30–45 min, 90% solvent B for 45–90 min, and 5% solvent B for 60–90 min. Survey scans in the 200–2,000 m/z range were performed with an MS resolution of 30,000 (at 400 m/z) in the Orbitrap analyzer, followed by 10 intensive precursor MS/MS scans by collision-induced dissociation at normalized collision energy (35%) [6].

### Database search, bioinformatics, and statistical analysis

All data were processed using the Uniprot bovine protein database (http://www.uniprot.org) and Proteome Discoverer 2.2 software. The identified protein contains at least one unique peptide per protein. In addition, the function and biological processes of the proteins in the data set were determined using gene ontology (GO) annotations. Determination of the signaling pathways involved in seminal plasma using PANTHER and the David database (https://david.ncifcrf.gov/). The analysis of interactions between proteins was carried out using STRING (http://string-db.org). Statistical analysis on semen parameters and protein intensity was performed using SPSS version 26 software.

## Results and Discussion

Data analysis revealed that the average quality of fresh Simmental semen in each of the NFS and PFS bulls was 70.00% ± 0.17% and 5.59% ± 0.07% sperm motility, 1,633.14 × 10^6^ cells/ml and 184.80 ×10^6^ cells/ml sperm concentrations, respectively (Table 1).

**Figure 1.**
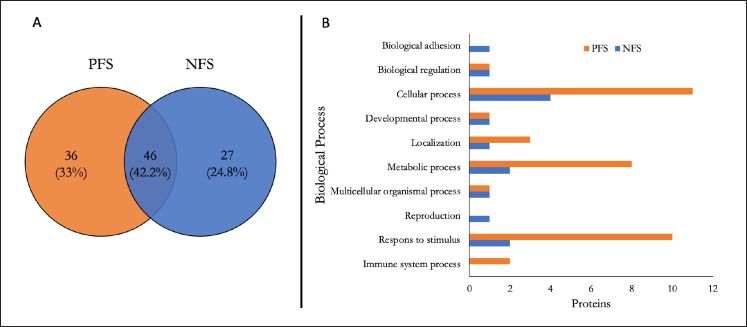
Proteins distribution: (A) Venn analysis (https://bioinfogp.cnb.csic.es/tools/venny/); normal bull (blue), infertile bull (yellow) and (B) proteins based on biological process (http://www.pantherdb).

According to the results of LC-MS/MS analysis, 73 proteins (seminal plasma NFS) and 82 proteins (seminal plasma PFS) were identified. Annotation analysis (Venn diagram) revealed that 46 proteins (42.2%) were found in both bovine seminal plasma (NFS and PFS). 27 proteins (24.8%) were found only in NFS, and 36 proteins (33%) were found in PFS (Fig. 1A). The results of this annotation analysis also show that NFS leads to both reproductive and adhesion processes, whereas PFS leads to the immune system process (Fig. 1B).

**Figure 2.**
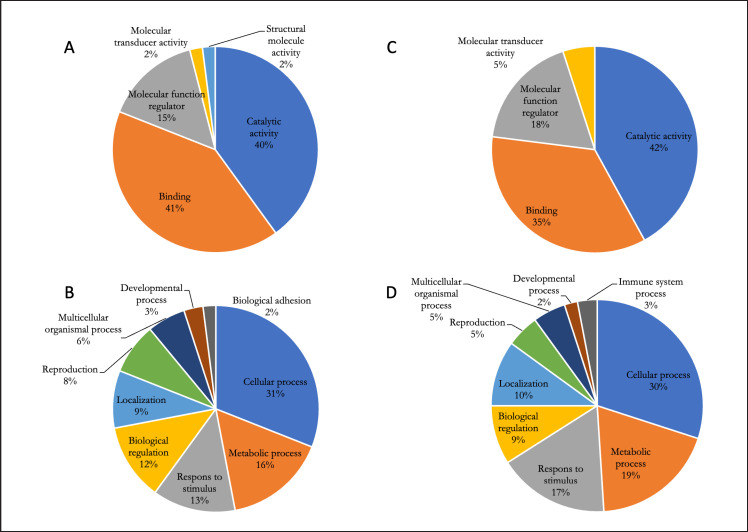
GO term Simmental bull normal (NFS) and infertile (PFS): (A) molecular function NFS; (B) biological process NFS; (C) molecular function PFS; and (D) biological process PFS (http://www.pantherdb).

Protein classification is based on two aspects of GO: molecular function and biological processes. The ontology analysis revealed that most proteins in NFS and PFS were directed towards a binding function, a catalytic activity, a regulator of a molecular function, a molecular transducer activity, and a structural activity (Fig. 2A and C). The results of GO analysis of biological processes in NFS and PFS showed that each protein was involved in a cellular process, a metabolic process, a response to stimulus, a biological regulation, a localization, a reproduction, a multicellular organismal process, a developmental process, and a biological adhesion (Fig. 2B and C). However, the results of the analysis indicate that the expression of plasma proteins in sperm leads to biological adhesion (NFS) and immune system processes (PFS).

**Table 1.** Sperm parameters Simmental bulls normal and infertile.

Parameter	NFS (60 × ejaculate)	PFS (76 × ejaculate)
Sperm motility (%)	70.00 ± 0.17^a^	5.59 ± 0.07^b^
Sperm concentration (×10^6^ ml^−^)	1,633.14 ± 31.78^a^	184.80 ± 24.41^b^

Means in a line with different superscripts a and b differ significantly at *p* < 0.05.

NFS: normal fresh semen; PFS: poor fresh semen/infertile.

The total protein identified in both NFS and PFS showed different protein characteristics. Generally, biological processes (cellular processes, regulation, and interactions between cells) and molecular functions (binding, catalytic activity) in seminal plasma are associated with events such as sperm motility, capacitation, acrosome reactions, fertilization, and embryonic development.

According to this study, there were cases of infertility associated with proteins leading to biological processes related to the immune system and antigens in the seminal plasma of PFS. A total of 36 different types of proteins (differentially expressed) were found in the seminal plasma of PFS bulls, 14 of which were involved in immunological processes as well as antigens. The protein mechanism identified by david.ncifcrf.gov/analysis leads to protein antigens via the complement cascade pathway, antigen processing, and apoptotic signaling via the PI3K-AKT signaling pathway.

**Figure 3.**
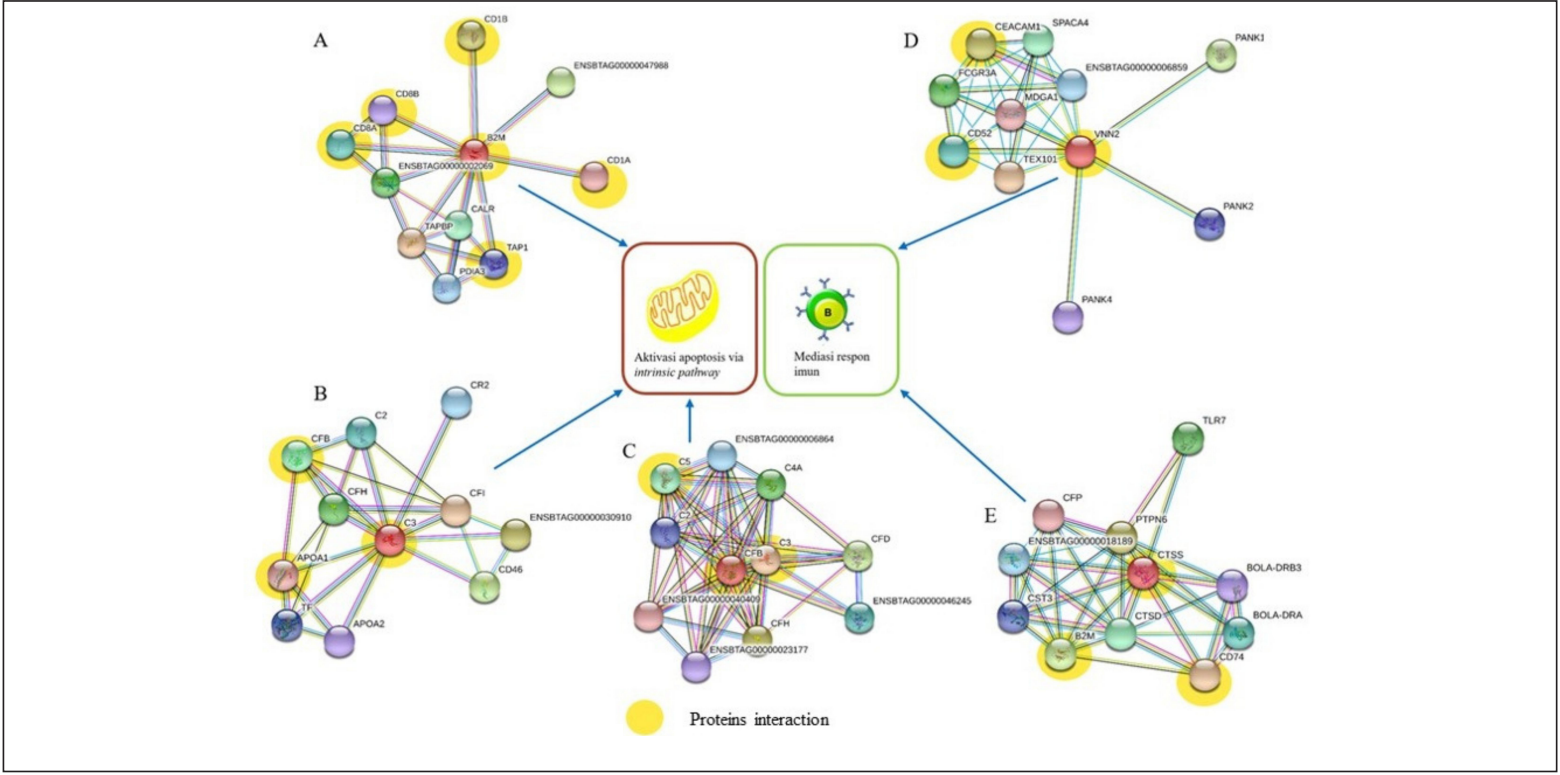
Interactions between seminal plasma proteins and reproductive function in infertile/PFS Simmental bulls (yellow): (A) B2M; (B) C3; (C) CFB; (D) VNN2; and (E) CTSS (STRING platform: http://string-db.org).

In the complement cascade pathway, the antigen–antibody complex activates complement-1qrs (C1qrs), which can convert C4b2a to C3 with the enzyme C3 convertase. Complement (C3) in the form of C3a and C3b changed from C42a3b to C5 with the help of the enzyme C5 convertase. Activation of C5 in the form of C5a and C5b can cause cell membrane damage (C5b-9), leading to cell lysis, including sperm. The complement system is one mechanism that Sertoli cells must modulate to prevent spermatocyte destruction [9]. In the regulation of the cascade pathway, protein C3 interacts with apolipoprotein A1 (APOA1) and complement factor B (CFB) (Fig. 3B and C).

The mechanism of the PI3K-AKT pathway can damage sperm membranes by DNA fragmentation through the activation of caspase signaling in Sertoli cells caused by a decrease in follicle stimulating hormone [12], which is associated with the expression of C3 that can interact with APOA1 in PFS seminal plasma. Iskandar et al. [1] reported that the presence of APOA1 in the seminal plasma of Bali cattle (*Bos javanicus*) may be negatively correlated with the percentage of normal sperm morphology and motility. APOA1 is a component of the high-density lipoprotein complex that can interact with proteins on the flagella and acrosomes of sperm [13]. The low motility of sperm in PFS bulls (5.59% ± 0.07%), presumably due to an interaction between APOA1 and proteins in the postacrosomal region and neck of sperm, may affect cholesterol efflux and inhibit fertilization in humans [14]. Moreover, the expression of the APOA1 antibody increases sperm apoptosis and decreases sperm motility [14].

The low sperm quality in PFS bulls is related to the expression of the protein beta-2-microglobulin (B2M), which can interact with cathepsin S/CTSS protein as an antigen (Fig. 3E), both as cytosolic antigens and endocytosed antigens. The role of B2M in antigen processing is mediated by the mechanisms of the MHC-I and MHC-II pathways. B2M activity in the MHC-I pathway is activated through two pathways: the interaction between the proteasome and the HSP70 family 13/HSP90 protein, which can activate TAP1/2 and MHC-I, and B2M, which plays a role in target cell damage through the T-cell receptor signaling pathway (CD8 T-cell via CD8 receptors and TCR) (Fig. 3A). The MHC-II pathway occurs in the endosome via the activation of CTSB/IL6 through the CD4 receptor and TCR through the T-cell receptor signaling pathway, which can stimulate cytokine production (regulation of the immune system) and cause a decrease in sperm quality in PFS bulls. Prihatno et al. [15] reported that IL6 is associated with testicular dysfunction, which affects the low semen quality of PFS bulls.

Expression of B2M and venin-2 (VNN2) proteins caused an increase in cytokines (IL6 and IL8) in PFS bulls. The presence of cytokines in the seminal plasma correlates with lower semen volume and sperm motility in bulls with reproductive tract dysfunction, resulting in low fertility [16]. The low quality of semen from PFS bulls was indicated to be related to the presence of growth factor (GF) and cytokines in seminal plasma. PI3K is activated by the interaction of GF-RTK, BCR-CD19, and cytokine-JAK, which can then activate AKT via PI3kinase stimulation. AKT activation can modulate multiple cellular pathways leading to cell proliferation and apoptosis and decreasing sperm concentration (azoospermia) and thus sperm quality [16]. In addition to the AKT and ERK signaling pathways that can regulate the phosphorylation processes, their activity could lead to changes in sperm velocity and even induce sperm apoptosis [17]. Furthermore, decreased sperm count (azoospermia) and motility were caused by the activation of cisplatin, which inhibits the AKT signaling pathway and induces apoptosis by increasing cleaved-caspase 3.

**Figure 4.**
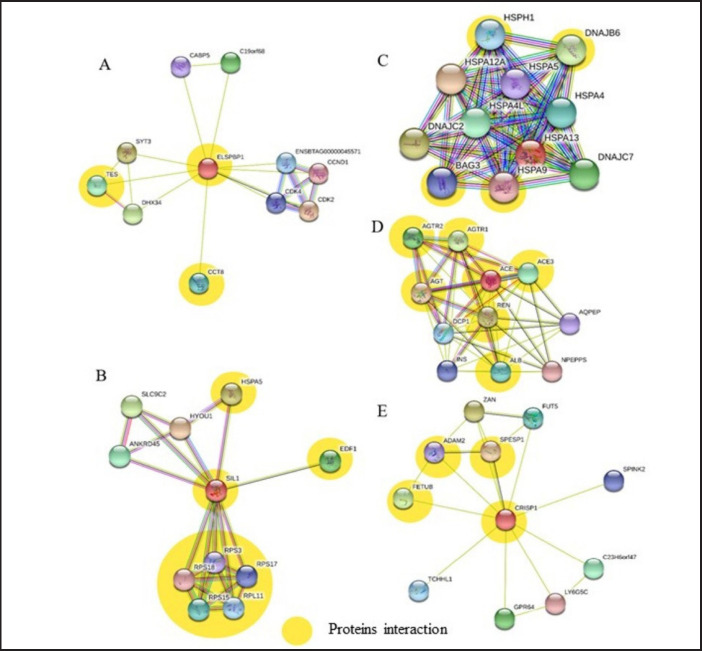
Interactions between seminal plasma proteins and reproductive function in Simmental bulls with NFS (yellow): (A) ELSPBP1; (B) SIL1; (C) HSPA13; (D) ACE; and (E) CRISP1 (STRING platform: http://string-db.org).

The STRING platform analysis revealed that epididymal sperm-binding protein 1 (ELSPBP1) (Fig. 4A) plays a role in reproductive function by initiating the process of sperm capacitation through the heparin-binding mechanism. ELSPBP1 is a sperm-binding protein that contains fibronectin type 2 (Fn_2_) and is found in the seminal plasma of buffalo sperm [18]. The function of ELSPBP1 is not known, but its interaction with binding sperm proteins shows that the two proteins bind to the phospholipid choline of the spermatozoa membrane during ejaculation and simultaneously regulate the Fn_2_ domain [18]. However, the expression of the two proteins shows different roles in the physiological function of sperm. Heparin interacts with BSPs and ELSPBP1 and may play a role in spermatozoa capacitation via heparin banding. Heparin binds to BSPs, decreasing their concentration and cholesterol and phospholipid levels in the plasma membrane [7]. Associated with the decreased induction of heparin are intracellular changes that initiate the release of Ca^2+^ via calcium ATPase. When the acrosome reaction occurs, Ca^2+^ increases. Heparin increase causes H^+^ efflux and HCO_3_− influx. The exchange of HCO_3_− and H^+^ raises intracellular pH (pHi) and activates soluble adenylate cyclase (sAC). Cyclic adenosine monophosphate production activates protein kinase A. It initiates signaling that can stimulate protein tyrosine kinase and inhibit protein tyrosine phosphatase, thereby increasing protein tyrosine phosphorylation, which is required for capacitation and acrosomal reactions [13].

The results of the analysis showed that the expression of protein nucleotide exchange factor (SIL1) (Fig. 4B) in sperminal plasma plays a role in protein transport and facilitates nucleotide exchange reactions in the endoplasmic reticulum [19]. SIL1 cooperates with the glucose-regulated protein of 170 kDa (Grp170) in modulating nucleotide exchange via binding between SIL1 in the nucleotide-binding domain (NBD) and immunoglobulin heavy-chain binding protein (BiP) [20]. NEF SIL1 is a single domain consisting of four substrates (Ia, Ib, IIa, and IIb) that bind to the lobes of BiP NBD. Upon binding of SIL1 in the form of BiP-adenosine diphosphate (ADP), the NBD of BiP is unlocked by the activity of lobe IIb, and ADP is released due to the instability in lobe Ib. Adenosine triphosphate (ATP) can then bind to NBD and BiP and trigger ATPase cycles, which are critical for protein activation and transport [19]. According to STRING platform analysis (Fig. 4C), HSP70 family proteins, including HSPA13, cooperate with SIL1 (BiP) to bind and release proteins, regulating ATP-binding, hydrolysis, and nucleotide exchange [21]. The binding of HSP70 to BiP initiates the hydrolysis of ATP to ADP. The release of ADP allows the rebinding of ATP to the substrate-binding domain, which is essential in nucleotide exchange reactions [19]. In addition, intracellular adenosine ATP is required for sperm movement and hyperactivation in the processes of capacitation and acrosomal responses, both of which are necessary for successful fertilization. However, Viana et al. [22] discovered that SIL1 protein was negatively associated with dairy bulls. The results of this study suggest that there is an interaction between SIL1 and HSPA13 that may reduce sperm motility via an ATP-binding mechanism.

According to the findings of this study, the angiotensin-1 covering enzyme (ACE) protein is one of the proteins in NFS associated with sperm reproductive function. The role and function of ACE could be in the regulation of steroidogenesis or Leydig cell function [23]. Karnik et al. [24] discovered that Ang-(1–7) is expressed in the testes as a ligand for the G-protein-coupled receptor Mas receptor on cell membranes. Mas mRNA in the testis is located in Leydig and Sertoli cells, which affects the expression of enzymes involved in testosterone biosynthesis in Leydig cells [23]. Pan et al. [25] found that ACE1, consisting of somatic ACE1 and testicular ACE1 (tACE1) isoforms, plays an important role in fertility and is expressed in the seminal plasma to protect sperm before and after deposition into the female reproductive tract. STRING platform analysis revealed that the ACE protein interacted with several proteins, including renin (RNN), angiotensin (Ang), ACE3, and albumin (Fig. 4D). The interaction of ACE1 (tACE1) and ACE3 regulates both spermatogenesis and fertilization [26].

The molecular mechanism involved in sperm fertilization ability through the interaction between proteins from the epididymis and the plasma membrane of sperm is played by cysteine-rich secretory protein (CRISP) [27]. In mammals, CRISP is expressed in four forms, namely CRISP1, CRISP2, CRISP3, and CRISP4, which are found in both reproductive and non-reproductive organs [28]. The expression of CRISP1 (Fig. 4E) in the semen plasma of NFS enables interaction with sperm via CRISP-sperm binding when transported from the male and female reproductive organs [27]. Ernesto et al. [28] found that CRISP1 plays a role in modulating sperm hyperactivation and participates in the regulation of several capacitation-associated events [27].

In addition, the presence of CRISP1 can inhibit TRPM8 and CatSper [28], which play a role in regulating sperm Ca^2+^ released from intracellular stores at the neck to prevent hyperactivation [29]. In this study, cases of infertility were found to be associated with the expression of proteins leading to biological processes related to the immune system and antigens in the seminal plasma of PFS beef bulls.

## Conclusion

There is an interaction between proteins in the seminal plasma of males with poor semen quality (PFS) and cases of infertility. A total of five proteins (PFS males) have been used as potential markers of male infertility, namely B2M, complement-3 (C3), CFB, VNN2, and CTSS.

## List of abbreviations

ADP, adenosine diphosphate; ATP, adenosine triphosphate; AIC, Artificial Insemination Center; BTB, blood-testicular barrier; BSE, breeding soundness examination; DTT, dithiothreitol; GO, gene ontology; LC-MS/MS, liquid chromatography-mass spectrometry; NFS, normal fresh semen; PFS, poor fresh semen; sAC, soluble adenylate cyclase; TEAB, triethylammonium bicarbonate.

## References

[1] Iskandar H, Andersson G, Sonjaya H, Arifiantini RI, Said S, Hasbi MT (2023). Protein identification of seminal plasma in Bali bull (*Bos javanicus*). Animals.

[2] Ashwitha A, Ramesha KP, Ramesh P, Kootimole CN, Devadasan MJ, Ammankallu S (2022). Quantitative proteomics profiling of spermatozoa and seminal plasma reveals proteins associated with semen quality in *Bos indicus* bulls. J Proteom.

[3] Baharun A, Arifinatini RI, Said S, Karja NWK (2021). Correlation between age, testosterone and adiponectin concentrations, and sperm abnormalities in Simmental bulls. Vet World.

[4] Indriastuti R, Ulum MF, Arifiantini RI, Purwantara B (2020). Individual variation in fresh and frozen semen of Bali bulls (*Bos sondaicus*). Vet World.

[5] Narwade B, Mohanty T, Bhakat M, Singh A, Rahim A, Sinha R (2018). Seasonal influence on semen production performance of crossbred buck (Saanen x Beetal) in an organized farm. Int J Livest Res.

[6] Fu Q, Pan L, Huang D, Wang Z, Hou Z, Zhang (2019). Proteomic profiles of buffalo spermatozoa and seminal plasma. Theriogenology.

[7] Diansyah AM, Yusuf M, Toleng AL, Ihsan M, Dagong A, Maulana T (2023). The sperm post-thawing quality and proteomic seminal plasma on fertility performance of Bali-polled bull. Adv Anim Vet Sci.

[8] Tanga BM, Qamar AY, Raza S, Bang S, Fang X, Yoon K (2021). Semen evaluation: methodological advancements in sperm quality-spesific fertility assessment—a review. Anim Biosci.

[9] Washburn RL, Hibler T, Kaur G, Dofour JM (2022). Sertoli cell immune regulation: a double-edged sword. Front Immunol.

[10] Washburn RL, Hibler T, Thompson LA, Kaur G, Dufour JM (2021). Therapeutic application of Sertoli cells for treatment of various diseases. Cell Dev Biol.

[11] Harrison TD, Chaney EM, Brandt KJ, Ault-Seay TB, Payton RR, Schneider LG (2022). The effects of nutritional level and body condition score on cytokines in seminal plasma of beef bulls. Front Anim Sci.

[12] Zhang H, Huang H, Zheng P, Feng R, Wang X, Huang F (2022). The alleviative effect of thyroid hormone on cold stress-induced apoptosis via HSP70 and mitochondrial apoptosis signal pathway in bovine Sertoli cells. Cryobiology.

[13] Benko F, Mohammadi-Sangcheshmeh A, Ďuračka M, Lukáč N, Tvrdá E (2022). *In vitro* versus cryo-induced capacitation of bovine spermatozoa, part 1: structural, functional, and oxidative similarities and differences. PLoS One.

[14] Chi X, Xiang D, Sha Y, Liang S, Wang C (2022). Inhibition of human sperm function by an antibody against apolipoprotein A1: a protein located in human spermatozoa. Andrologia.

[15] Prihatno SA, Adi YK, Budipitojo T (2020). Immunolocalization of IL-6 and IL-10 in the testicular tissue of testicular dysfuntion rat treated with secretome. J Adv Vet Anim Res.

[16] Li X, Luo T, Li H, Yan N (2020). Sphingomyelin synthesa 2 participate in the regulation of sperm motility and apoptosis. Molecules.

[17] Shati AA (2019). Resveratrol improves sperm parameter and testiscular apoptosis in cisplatin-treated rats: effects on ERK1/2, JNK, and Akt pathways. Syst Biol Rep Med.

[18] Kumaresan A, Sinha MK, Paul N, Nag P, King JPES, Kumar R (2023). Establishment of a repertoire of fertility associated sperm proteins and their differential abundance in buffalo bulls (*Bubalus bulalis*) with contrasting fertility. Nature.

[19] Pobre KFR, Poet GJ, Hendershot LM (2019). The endoplasmic reticulum (ER) chaperone BiP is a master regulator of ER funtions: getting by with a little help from ERdj friends. J Biol Chem.

[20] Conza GD, Ho PC, Cubillos-Ruiz JR, Huang SCC (2023). Control of immune cell function by the unfolded protein response. Nat Rev Immunol.

[21] Kityk R, Kopp J, Mayer MP (2018). Molecular mechanism of J-domain-triggered ATP hydrolysis by Hsp70 chaperones. Mol Cell.

[22] Viana AGA, Martins AMA, Pontes AH, Fontes W, Castro MS, Ricart CAO (2018). Proteomic landscape of seminal plasma associated with dairy bull fertility. Sci Rep.

[23] Pan PP, Zhan QT, Le F, Zheng YM, Jin F (2013). angiotensin-converting enzymes play a dominant role in fertility. Int J Mol Sci.

[24] Karnik SS, Singh KD, Tirupula K, Unal H (2017). significance of angiotensin 1-7 coupling with MAS1 receptor and other GPCRs to the renin-angiotensin system: IUPHAR review 22. Br J Pharmacol.

[25] Pan Y, Cui Y, Baloch AR, Fan J, He J, Li G (2015). Insulin-like growth factor I improve yak (*Bos grunniens*) spermatozoa motility and the oocyte cleavage rate by modulating the expression of Bax and Bcl-2. Theriogenology.

[26] Tumova L, Zigo M, Sutovsky P, Sedmikova M, Postlerova P (2021). Ligans and receptors involved in the sperm-zona pellucida interactions in mammals. Cells.

[27] Gonzalez SN, Sulzyk V, Mun`oz MW (2021). Cysteine-rich secretory proteins (CRISP) are key players in mammalian fertilization and fertility. Front Cell Dev Biol.

[28] Ernesto JI, Mun`oz MW, Battistone MA, Vasen G, Martınez-Lopez P, Orta G (2015). CRISP1 as a novel CatSper regulator that modulates sperm motility and orientation during fertilization. J Cell Biol.

[29] Rennhack A, Schiffer C, Brenker C, Fridmad D, Nitao ET, Cheng YM (2018). A novel cross-species inhibitor to study the function of CatSper Ca^2+^ channels in sperm. Br J Pharmacol.

